# Detection of cervical lymph node metastasis from oral cavity cancer using a non-radiating, noninvasive digital infrared thermal imaging system

**DOI:** 10.1038/s41598-018-24195-4

**Published:** 2018-05-08

**Authors:** Fan Dong, Chuansibo Tao, Ji Wu, Ying Su, Yuguang Wang, Yong Wang, Chuanbin Guo, Peijun Lyu

**Affiliations:** 1 National Engineering Laboratory for Digital and Material Technology of Stomatology, Peking University School and Hospital of Stomatology, 22 Zhongguancun Avenue South, Haidian District Beijing, 100081 P. R. China; 2 Center of Digital Dentistry, Peking University School and Hospital of Stomatology, 22 Zhongguancun Avenue South, Haidian District Beijing, 100081 P. R. China; 3 Department of Prosthodontics, Peking University School and Hospital of Stomatology, 22 Zhongguancun Avenue South, Haidian District Beijing, 100081 P. R. China; 4 Research Center of Engineering and Technology for Digital Dentistry of Ministry of Health, 22 Zhongguancun Avenue South, Haidian District Beijing, 100081 P. R. China; 5 Beijing Key Laboratory of Digital Stomatology, 22 Zhongguancun Avenue South, Haidian District Beijing, 100081 P. R. China; 6 Department of Oral and Maxillofacial Surgery, Peking University School and Hospital of Stomatology, 22 Zhongguancun Avenue South, Haidian District Beijing, 100081 P. R. China; 7 Tsinghua-Rohm Electronic Engineering Hall 8-301, Tsinghua University, Beijing, 100084 P. R. China

## Abstract

This study aimed to evaluate the diagnostic performance of a non-radiating, noninvasive infrared (IR) thermal imaging system in the detection of cervical lymph node metastasis from oral cavity cancer. In this prospective clinical trial, a total of 90 oral cavity cancer patients suspected of having cervical lymph node metastasis underwent IR imaging of the neck prior to neck dissection. Analysis of the IR images was performed by two methods: manual qualitative analysis and automatic analysis by an entropy-gradient support vector machine (EGSVM). The efficacies of the EGSVM-based infrared thermal imaging system and contrast-enhanced computed tomography (CT) were compared by using the Noninferiority Testing. Compared with manual qualitative analysis, the EGSVM-based automatic analysis had a higher sensitivity (84.8% vs. 71.7%), specificity (77.3% vs. 72.7%), accuracy (81.1% vs. 72.2%), positive predictive value (79.6% vs. 73.3%) and negative predictive value (82.9% vs. 71.1%). The EGSVM-based infrared thermal imaging system was noninferior to contrast-enhanced CT (*P* < 0.05). The EGSVM-based infrared thermal imaging system showed a trend of higher sensitivity, whereas contrast-enhanced CT showed a trend of higher specificity. The EGSVM-based infrared thermal imaging system is a promising non-radiating, noninvasive tool for the detection of lymph node metastasis from oral cavity cancer.

## Introduction

Oral cavity cancer (ICD-10:C00-C08) is a serious and growing problem in numerous countries. Worldwide, in 2012, there were approximately 300,400 new cases and 145,400 deaths from oral cavity cancer^1^. Controlled the primary tumor, metastasis to the cervical lymph node is the most significant factor that determines prognosis^2^. Regardless of the site of the primary tumor, the 5-year survival rate drops by nearly 50% if a single metastatic lymph node is present in either side of the neck. The survival rate of patients with a single metastatic lymph node in both sides is reduced to only 25% of that in patients without lymph node metastasis^3–5^. Therefore, assessment of lymph node involvement is of utmost importance in patients with oral cavity cancer.

The current diagnostic modalities for the detection of lymph node metastasis include computed tomography (CT), positron emission tomography/computed tomography (PET/CT), magnetic resonance imaging (MRI), ultrasound, and ultrasound-guided fine needle aspiration cytology (FNAC). Due to the superior anatomic resolution, CT and MRI are commonly used for the detection of cervical lymph node metastasis^6^. Each technique has its own unique advantages and disadvantages. In clinical practice, we must compromise between accuracy and effectiveness vs. invasiveness and cost considerations. An innocuous, noninvasive imaging modality remains an open quest in biomedical imaging. Among the current modalities mentioned above, only MRI and ultrasound do not involve radiation exposure or invasive procedures. Ultrasound is a widely available imaging modality but it is restricted to expert referral centers because its diagnostic performance is highly dependent on the experience of the operator^7^. With a relatively expensive cost, MRI is not widely available. Therefore, a reliable, new, cost-effective method is desired.

Infrared (IR) thermal imaging is a non-radiating, noncontact, noninvasive, low-cost and fast imaging modality that passively captures thermal radiation emitted by any object above absolute zero. Unlike other imaging modalities, IR imaging provides functional rather than anatomical information because temperature is a useful indicator of disease. Previous studies have found that temperature distribution is symmetrical between the two sides of the human body in healthy people; in diseased individuals, abnormal blood flow results in abnormal temperature distribution^8^. For instance, excess heat generated by blood flow (angiogenesis) and metabolic activity in breast cancer provide the basis for the detection of breast cancer with IR imaging^9^. With technological advances in thermal cameras and image analysis tools over the years, there has been a resurgence in the use of IR thermal imaging as a diagnostic tool in medicine^10^. IR imaging has been applied to the diagnosis of many diseases such as breast cancer^10,11^, melanoma^12^, diabetes^13^, infantile hemangiomas^14^ and lower extremity deep venous thrombosis^15^. To the best of our knowledge, IR imaging has not been applied to the detection of cervical lymph node metastasis.

Support vector machine (SVM) is a supervised machine learning technique that is widely used in classification problems. The algorithm uses special nonlinear functions called kernels to transform the input space into high-dimensional space and aims to select a hyperplane to discriminate between two classes by maximizing the margin between two data clusters^16^. The technique has recently been used to improve diagnostic performance^17,18^.

In this study, we proposed a digital infrared thermal imaging system as a screening and patient-based diagnostic tool for the detection of cervical lymph node metastasis from oral cavity cancer. Then, the analysis of the IR images was performed by two methods: manual qualitative analysis and automatic analysis by an entropy-gradient support vector machine (EGSVM), the classification was done on a per patient basis. We also investigated its diagnostic performance and compared it with that of contrast-enhanced CT which is now routinely used for the detection of cervical lymph node metastasis in Stomatology Hospital of Peking University.

## Methods

### Study design

**Figure 1:**
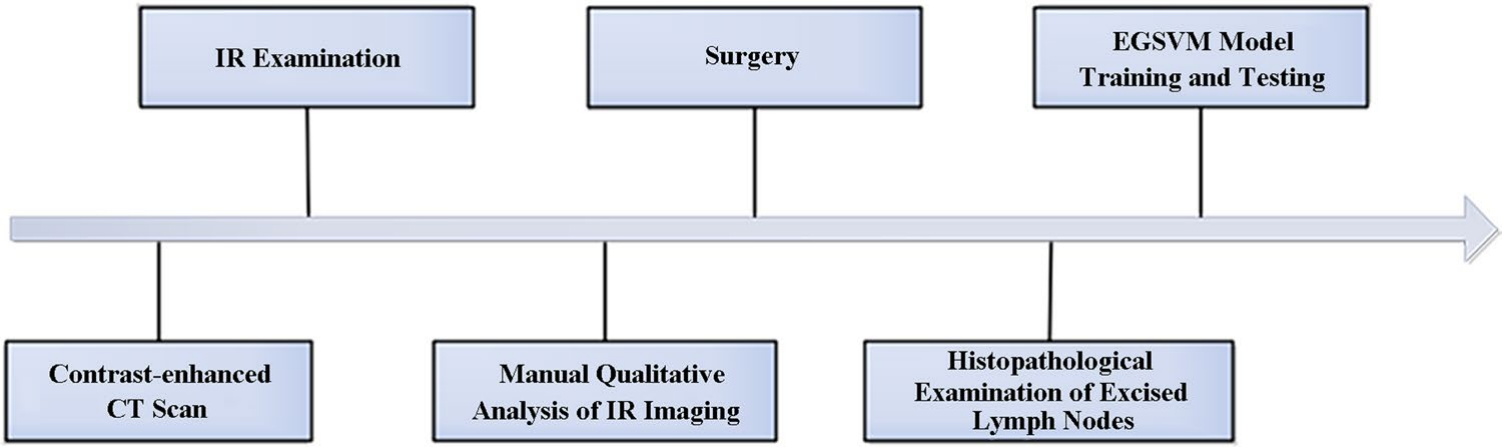
Timeline of the study process.

This prospective study was approved by the Bioethics Committee of Stomatology Hospital of Peking University, Beijing, China (No. PKUSSIRB-201628047), and all patients signed informed consent forms prior to entering the study. The study protocol is shown in Fig. 1. We enrolled a series of 90 patients (60 male [66.7%], 30 female [33.3%]), ranging from 29 to 81 years old (mean = 58.2 years, SD = 12.3 years) who were scheduled for neck dissection with resection of previously untreated primary oral cancer. We took no account of the result of IR examination when the treatment plan was made. Of the 90 patients in our study, 44 (48.9%) were clinically N0 (no suspected metastasis) based on physical exam and contrast-enhanced CT results before the surgery, while 46 (51.1%) were clinically N+ (suspected metastasis). The site and histological type of the primary tumor are provided in Table 1. Apart from the diagnostic biopsy of the primary tumor and previous routine dental treatment, no patients had undergone previous head and neck surgery, chemotherapy, or radiotherapy.

**Table 1:** Patient characteristics.

Title	Subtitle	Number	Percentage
Number of Patients		90	
Age (years)		58.2 ± 12.3	
Gender	Male	60	66.7%
	Female	30	33.3%
Clinical Examination	cN0	44	48.9%
	cN+	46	51.1%
**Site of the Primary Tumor**			
ICD10-C00	Lip	1	1.1%
ICD10-C01	Base of Tongue	7	7.8%
ICD10-C02	Tongue	31	34.5%
ICD10-C03	Gum	24	26.7%
ICD10-C04	Floor of Mouth	7	7.8%
ICD10-C05	Palate	1	1.1%
ICD10-C06.0	Buccal Mucosa	11	12.2%
ICD10-C06.2	Retromolar Area	4	4.4%
ICD10-C10	Oropharynx	4	4.4%
Historical Type			
	Squamous Cell Carcinoma	77	85.6%
	Mucosal Malignant Melanoma	6	6.7%
	Adenoid Cystic Carcinoma	3	3.3%
	Basal Cell Adenocarcinoma	1	1.1%
	Adenosquamous Cell Carcinoma	1	1.1%
	Sarcomatoid Carcinoma	1	1.1%
	Clear Cell Carcinoma	1	1.1%

### IR examination

IR imaging of the neck was performed 1 day before surgery. IR examination was performed by one radiological technician with a thermographic system (Avio R500 Thermal Imaging System, NEC Corporation, Japan), which was an uncooled micro-bolometer with a focal plane array detector. The image matrix size was 640 × 480, with a response wavelength of 8–14 μm, and a temperature resolution <0.025 °C. The procedure was performed in a temperature-controlled room maintained between 23 °C and 25 °C and 50% relative humidity. Each participant was asked to sit on a chair in an erect position, with the neck exposed, approximately 0.5 meters away from the IR camera. After 15 minutes of rest, IR images of the frontal neck were taken.

### Manual qualitative analysis of IR imaging

**Figure 2:**
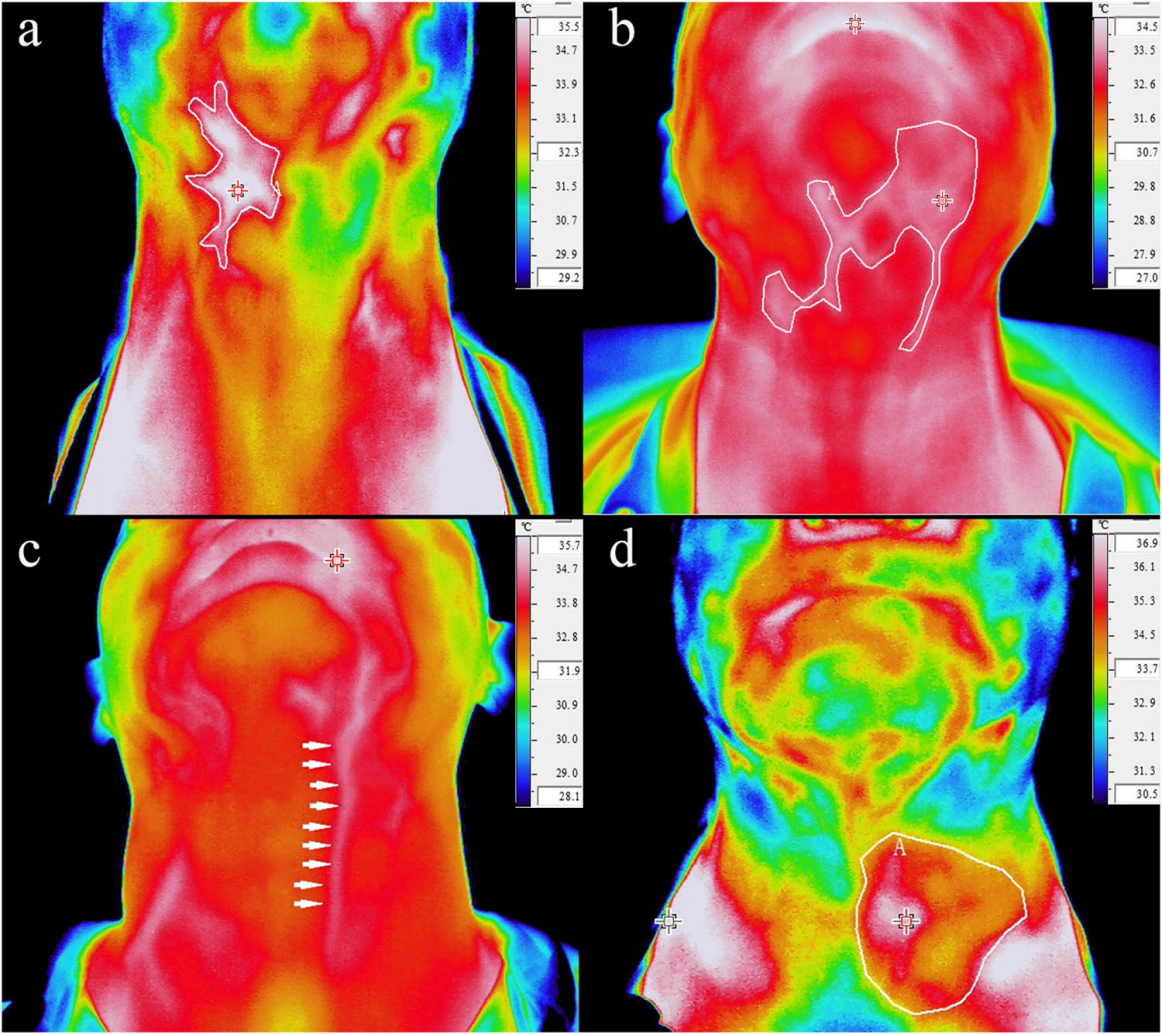
Manual qualitative analysis of IR imaging. (**a**) An asymmetric thermographic pattern that includes an elevated surface temperature and a vascular pattern; (**b**) Increased vascular density with a tortuous vascular morphologic pattern; (**c**) A unilateral dilated vascular image; (**d**) A surface temperature difference of over 1 °C.

Qualitative analysis was performed by two experienced head and neck radiologists who were unaware of the histological results and the contrast-enhanced CT results using Infrec Analyzer 2.6 software (NEC Corp., Japan) with manual brightness and contrast adjustment. Disagreements between two radiologists were resolved via consensus. In this study, IR criteria for the detection of metastasis were modified from those used in breast cancer^19,20^. The presence of at least one of the following criteria (Fig. 2) was considered a positive indicator for cervical lymph nodal metastasis: (a) increased vascular density with a tortuous vascular morphologic pattern or aberrant vasculature in the region of interest (ROI) but not in the contralateral side; (b) unilateral dilated vasculature such as a facial artery, a submental artery or a carotid artery; (c) a surface temperature difference >1 °C in the ROI compared to the mirror image site on the contralateral neck; (d) a bulging outline contour with elevated surface temperature in and around the ROI.

### Surgery and histopathological examination

The range of neck dissection was based on the criteria of Stomatology Hospital of Peking University. The indications and choice of a neck dissection were determined based on preoperative examination results and intraoperative findings. Patients with evidence of clinically N2 or N3^21^ underwent a radical neck dissection (RND), in which the scope of the surgery involved cervical lymph nodes level I, II, III, IV and V. The preservation of important anatomical structures, such as the internal jugular vein, the accessory nerve, and the sternocleidomastoid muscle, depends on the relationship between these anatomical structures and the suspected metastases observed during the surgery. Patients with evidence of clinically N1 underwent a selective neck dissection, such as supraomohyoid neck dissection (SOHND) or extended supraomohyoid neck dissection, where the scope of surgery involved cervical lymph nodes of level I-III or level I-IV, respectively. For malignancies with a high risk of cervical metastasis, a selective neck dissection was required even if the preoperative impression was cN0. The contralateral neck of a metastatic lymph node then underwent a selective neck dissection or functional neck dissection considering the risk of contralateral metastasis. Additionally, if the primary tumor crossed the midline, a bilateral neck dissection was also necessary.

At the time of surgery, the partitioned surgical neck specimens were separated by surgeons and fixed in 10% buffered formalin. The dissected lymph nodes were processed and stained with hematoxylin and eosin for pathological assessment. A routine pathological evaluation of the lymph nodes was performed by two pathologists on one or two sections^22^. Diagnoses were reviewed by one pathologist with 10 years of experience. All nodes were recorded as positive or negative for metastasis.

### Automatic analysis by EGSVM

Given a training set if instance-label pairs ${x}_{i},{y}_{i},i=1,\ldots ,m$ where ${x}_{i}\in {R}^{n}$ and $y\in {\{1,-1\}}^{n}$, the objective of SVM is to choose the optimal hyperplane that separates the instances into two groups, and maximizes the distance between the hyperplane and the support vectors^23,24^. In other words, the SVM aims to solve the optimization problem:

1 $${mi}{{n}}_{w,b,\varepsilon }\frac{1}{2}{w}^{T}w+C{\sum }_{i=1}^{m}{\varepsilon }_{i}$$

2 $${\rm{subject}}\,{\rm{to}}\,{y}_{i}({w}^{T}{\rm{\phi }}({x}_{i})+b)\ge 1-{\varepsilon }_{i}$$

where ${\varepsilon }_{i}\ge 0$. The function ${\rm{\phi }}$ maps feature vectors *x*_*i*_ into a higher dimensional space. *C* is a penalty parameter on the training error. A kernel function is written as $K({x}_{i},{x}_{j})={\rm{\phi }}{({x}_{i})}^{T}{\rm{\phi }}({x}_{j})$. In our experiment comparison, the linear kernel $K({x}_{i},{x}_{j})={x}_{i}^{T}{x}_{j}$ performs best in the proposed medical image classification task.

**Figure 3:**
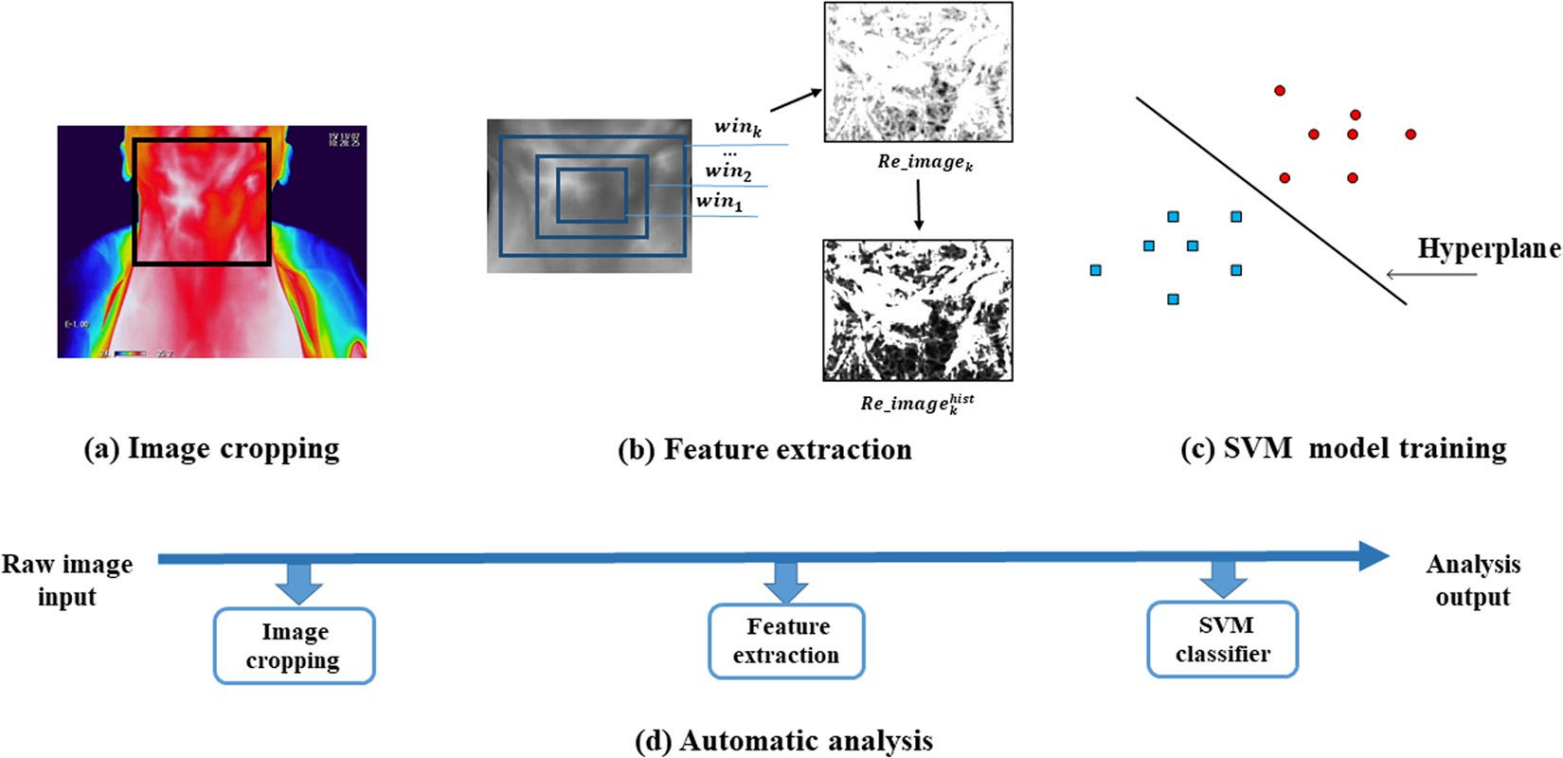
Structure of the EGSVM system. (**a**) The image in the black box is the cropped image from raw images, which is converted into a grayscale image; (**b**) The expanding process of *win* and feature extraction. The *win* expands from *win*_1_ to *win*_*n*_. For *win*_*k*_, the entropy of $Re\_imag{e}_{{\rm{k}}}$ and $\,Re\_imag{e}_{k}^{hist}$ are used in the feature extraction process; (**c**) The red circles and blue squares represent high-dimensional feature vectors; (**d**) The structure of automatic analysis system for new raw images.

The EGSVM is a four-step procedure (Fig. 3). In the first step (Fig. 3a), the region of the neck is cropped from raw images, which are then converted to grayscale images. The target area is from the lower jaw to the clavicles included in the cropped image. The background and unrelated parts in the raw image are cut out. One cropped image is acquired from each raw image manually. Second, features used for classification are extracted from the cropped grayscale images (Fig. 3b). The feature vectors are fed to an SVM classifier in the third step (Fig. 3c). Model parameters of an SVM classifier are trained on the features and corresponding labels. Finally, the structure (Fig. 3d) is used for the automatic analysis of lymph node involvement.

#### Feature extraction

During manual qualitative analysis, we found that an asymmetric thermographic pattern, including elevated surface temperature and abnormal vascular pattern, was an important indication of nodal involvement (Table 2). Hence, features that can describe an irregularity of an IR image would be helpful to distinguish metastatic lymph images from the other images. During image processing, entropy is a commonly used measure of information contained in an image^25,26^. In the feature extraction process, the cropped grayscale image is first normalized according to its window size as follows:

3 $$gVal\_new=gVal(x,y)\cdot \frac{255}{{ma}{{x}}_{(x^{\prime} ,y^{\prime} )\in win}\,gVal(x^{\prime} ,y^{\prime} )}$$

where *win* represents a window in the original image. $gVal\_new(x,y)$ is the gray value at point (*x*, *y*) of the normalized image, which is obtained by scaling the gray value of the corresponding point $gVal(x^{\prime} ,y^{\prime} )$ in the original image.

**Table 2:** Distribution of patient cases by IR features.

IR Features	No. of Cases	No. of Metastatic Cases	Prevalence (%)
Abnormal vascular morphology	29	21	72.4
Unilateral dilated vessel	10	7	70.0
Temperature difference over 1 °C	10	7	70.0
Focal bulge	3	3	100.0

Prevalence = number of metastatic cases divided by number of cases with the given IR features.

If we change the size of window, we can obtain a series of normalized images with different sizes, denoted $\{Re\_imag{e}_{1},Re\_imag{e}_{2},\ldots ,Re\_imag{e}_{n}\}$, where *n* represents the quantity of all normalized images. The extending process of *win* and feature extraction process are shown in Fig. 3c. In our experimental settings, *win* extends from central to around evenly.

Histogram equalization is a technique to enhance contrast by adjusting the image spatial domain. The technique produces output image with uniform distribution of pixel intensity, flattened histogram^27^. To enhance the contrast of normalized images, we acquire a histogram-equalized image of each of them, denoted $\{Re\_imag{e}_{1}^{hist},Re\_imag{e}_{2}^{hist},\ldots ,Re\_imag{e}_{n}^{hist}\}$. The extending step is set (1/50 of width, 1/50 of height) in our feature extraction stage. Therefore, *n* is 50 in our experiments. For each image $Re\_imag{e}_{k}$, we can calculate the corresponding entropy *h*_*k*_ as

4 $${h}_{k}=-\,{\sum }_{i=1}^{255}{p}_{i}\,\mathrm{log}\,{p}_{i},\,k\in [1,n]$$

where *p*_*i*_ is the proportion of gray level *i* in $Re\_imag{e}_{k}$, and these entropies can make up for a vector $H=[{h}_{1},{h}_{2},\ldots ,{h}_{n}]$. We can further get the entropy-gradient feature ${H}_{var}$, based on *H*, as

5 $${H}_{var}=[{h}_{2}-{h}_{1},\ldots ,{h}_{n}-{h}_{n-1}]$$

The entropy-gradient feature ${H}_{var}^{hist}$ is extracted from histogram equalized image sets in the same way as ${H}_{var}$ of the set of normalized images.

One disadvantage of histogram equalization is that the calculation is indiscriminate. The gray value of the histogram equalized image is non-linearly stretched from that in the cropped gray image. It increases the image contrast while decreases the usable information. Different from histogram image, the gray value of normalized image is linearly stretched from that in the cropped image, preserving more details with little contrast. Considering the advantages and disadvantages of both images, $[{H}_{var},{H}_{var}^{hist}]$ is finally used as the feature vector for the input of the SVM classifier.

#### Model training and prediction

We used an SVM from libsvm tool^23^ to complete our experiments. The feature vectors acquired from images with lymphoma were assigned weight 1.2 while others were assigned weight 1.0. The penalty parameter of the error term *C* was set as 0.5. As previously mentioned, there were 90 samples in total. To make better use of these data, a nine-fold cross-validation method was applied in the testing. The dataset was randomly divided into nine subsets, each containing an equal number of samples. The nine subsets were then grouped into a training set and a testing set. The training set consisted of eight of these subsets and the testing set consisted of the remaining one. This procedure was repeated nine times and every subset was used once for testing. The final matrices were the average of the five testing results, including sensitivity, specificity, accuracy, positive predictive value (PPV) and negative predictive value (NPV).

### Statistical analysis

All statistical analyses were performed in R version 3.1.0. Based on the standard definitions, sensitivity, specificity, accuracy, PPV and NPV were calculated. The accuracies of the EGSVM-based infrared thermal imaging system and contrast-enhanced CT for the detection of cervical lymph node metastasis from oral cavity cancer were compared by using the Noninferiority Testing^28^, and the value of margin was set at δ = 0.10, and *P* values less than 0.05 were considered statistically significant.

### Ethical approval

This prospective study was approved by the Bioethics Committee of Stomatology Hospital of Peking University, Beijing, China (No. PKUSSIRB-201628047). All experimental protocols were approved by the Bioethics Committee of Stomatology Hospital of Peking University, Beijing, China. All experiments were performed in accordance with approved guidelines and regulations. Informed consent was obtained from all individual participants included in the study.

## Results

In our study, tissues from a total of 90 patients were excised for histopathological correlation. Lymph node metastasis was present in 46 (51.1%) patients, while the other 44 (48.9%) patients did not contain histological evidence of lymph node metastasis. Of the 46 clinically N+ patients, 34 (73.9%) contained histological evidence of lymph node metastasis and the remaining 12 (26.1%) did not. Among the 44 clinically N0 patients, 12 (27.3%) contained histological evidence of lymph node metastasis, and the remaining 32 (72.7%) did not.

Based on IR criteria for manual qualitative analysis (Fig. 2), the distribution of patient cases is shown in Table 2. The sensitivity, specificity, accuracy, PPV and NPV by manual qualitative analysis for the detection of lymph node metastasis were 71.7% (95% CI: 58.7%, 84.8%), 72.7% (95% CI: 59.6%, 85.9%), 72.2% (95% CI: 63.0%, 81.5%), 73.3% (95% CI: 60.4%, 86.3%), and 71.1% (95% CI: 57.9%, 84.4%), respectively. Compared with manual qualitative analysis, EGSVM-based automatic analysis had an apparently higher sensitivity (84.8% vs. 71.7%), specificity (77.3% vs. 72.7%), accuracy (81.1% vs. 72.2%), PPV (79.6% vs. 73.3%) and NPV (82.9% vs. 71.1%) (Table 3).

**Table 3:** Performance of manual and automatic analysis of IR imaging.

Parameters	Manual qualitative analysis	Automatic analysis by EGSVM
No. of true positive	33	39
No. of false positive	12	10
No. of true negative	32	34
No. of false negative	13	7
Sensitivity (%)	71.7 (58.7,84.8)	84.8 (74.4,95.2)
Specificity (%)	72.7 (59.6,85.9)	77.3 (64.9,89.7)
Accuracy (%)	72.2 (63.0,81.5)	81.1 (73.0,89.2)
PPV (%)	73.3 (60.4,86.3)	79.6 (68.3,90.9)
NPV (%)	71.1 (57.9,84.4)	82.9 (71.4,94.4)

Data in parentheses are 95% CIs; Sensitivity = true positive/(true positive + false negative); Specificity = true negative/(true negative + false positive); Accuracy = (true positive + true negative)/(true positive + true negative + false positive + false negative); PPV = true positive/(true positive + false positive); NPV = true negative/(true negative + false negative).

The EGSVM-based infrared thermal imaging system is noninferior to contrast-enhanced CT (*P* = 0.01), and the statistical power in this trial is 80.34% (δ = 0.10). The EGSVM-based infrared thermal imaging system showed a trend of higher sensitivity, whereas contrast-enhanced CT showed a trend of higher specificity (Table 4).

**Table 4:** Comparison of diagnostic efficacies between contrast-enhanced CT and the EGSVM-based infrared thermal imaging system.

	No. of patients with metastasis			No. of patients without metastasis		
	CT+	CT−	Total	CT+	CT−	Total
EGSVM+	29	10	39	1	9	10
EGSVM−	5	2	7	6	28	34
Total	34	12	46	7	37	44
Group	Sen (%)	Spe (%)	Accuracy (%)	Youden’s index	LR+	LR−
CT	73.9 (61.2, 86.6)	84.1 (73.3, 94.9)	78.9 (70.5, 87.3)	0.580	4.6478	0.3103
EGSVM	84.8 (74.4, 95.2)	77.3 (64.9, 89.7)	81.1 (73.0, 89.2)	0.621	3.7357	0.1966

Data in parentheses are 95% CIs; CT+ = positive at contrast-enhanced CT; CT− = negative at contrast-enhanced CT; EGSVM+ = positive at EGSVM-based infrared thermal imaging system; EGSVM− = negative at EGSVM-based infrared thermal imaging system; Sen = sensitivity; Spe = specificity; LR+ = positive likelihood ratio; LR− = negative likelihood ratio.

**Figure 4:**
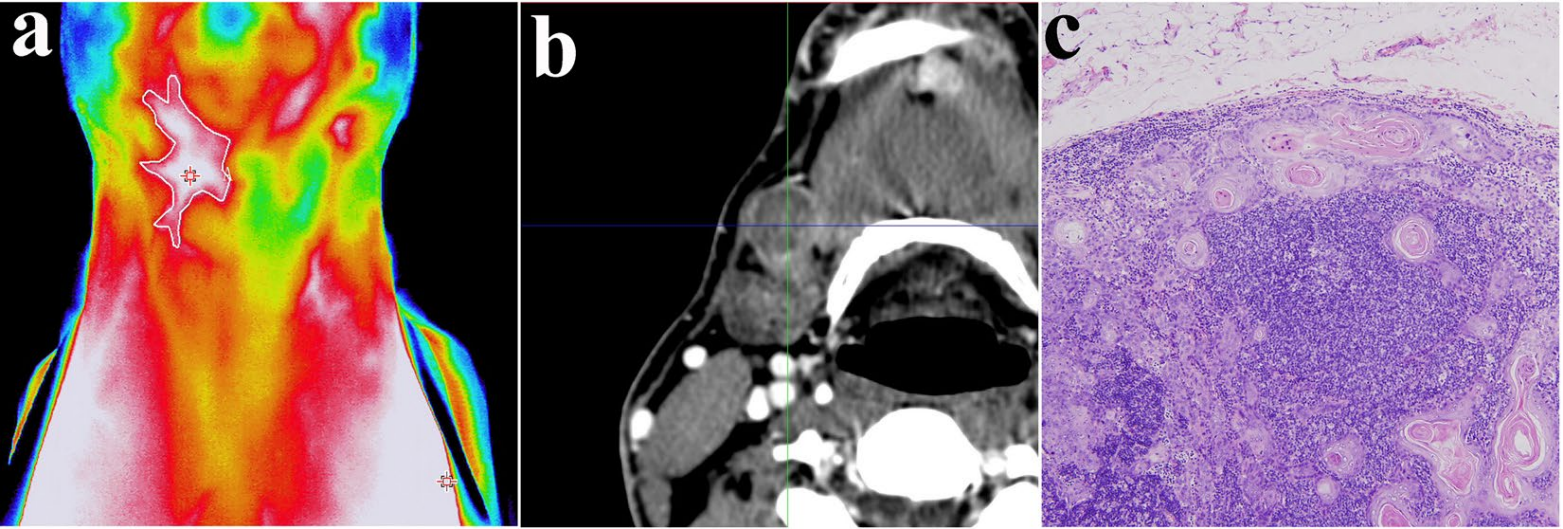
Representative case correctly confirmed with both IR imaging and contrast-enhanced CT. (**a**) Elevated surface temperature and increased vascular density on the right side using IR imaging; (**b**) Metastatic lymph node in contrast-enhanced CT; (**c**) Microscopic appearance of metastatic lymph nodes (hematoxylin and eosin staining, original magnification ×200).

Among the 46 patients with metastasis, 29 cases were correctly confirmed with both contrast-enhanced CT and the EGSVM-based infrared thermal imaging (Fig. 4); only 2 cases were not confirmed with either enhanced CT or the EGSVM-based infrared thermal imaging because of occult metastasis. Of the 44 patients without metastasis, 28 cases were not confirmed with either enhanced CT or the EGSVM-based infrared thermal imaging; only one case was confirmed with these two imaging modalities, resulting in a false positive (Table 4).

## Discussion

In this prospective study, we performed the first application of digital infrared thermal imaging to the detection of cervical lymph node metastasis from oral cavity cancer. We also found that the EGSVM-based infrared thermal imaging system is objective and reliable as a screening and diagnostic tool for the detection of cervical lymph node metastasis from oral cancer.

With manual qualitative analysis, we found that high frequency tumor-associated vascular abnormalities are powerful indicators of nodal involvement (Table 2). Compared with vessels in healthy tissues, tumor-associated vessels have long been observed to be abnormal in terms of morphology and structure, even at early stages of disease^29^. In animal models, tumor-associated tortuous vessel morphologies appeared much earlier than a palpable mass, even when only tens of tumor cells were introduced into the tissue^30^. As tumor growth needs an ever-increasing nutrient supply, malignant tumors generate a unique vessel system by creating new vessels (angiogenesis) and influencing major vessels around the tumor via angiogenic growth factors^31^. Therefore, the progression of angiogenesis over time has been imaged in an animal model of lymph node metastasis^32^. The tumor-associated vascular abnormalities we found in manual qualitative analysis, including increased vascular density with a tortuous vascular morphologic pattern, aberrant vasculature and a unilateral dilated vascular pattern in IR images, were consistent with previous studies. The metabolic activity and abnormal vessel pattern resulted in deviations in heat. It is this deviation in heat that provides the basis for the use of infrared imaging.

EGSVM is another major part of our work that contributes to the diagnostic performance. We have proposed an efficient computer-aided diagnosis system that uses the EGSVM model for classification. Computer-aided diagnostics have been studied in various diseases via medical images^33^. A good computer-aided diagnosis system can eliminate operator dependency, improve diagnostic performance, and reduce the time needed for the interpretation of images. During manual qualitative analysis, abnormal signs are based on only relatively large areas that are visible to the naked eye, and thus, small lesions may be ignored. Automatic classification systems can describe the irregularity of images, allowing for greater objectivity in image processing and reduced inter-observer variability. In addition, the automatic analysis of images could be completed in under a minute, greatly improving efficiency. Due to the satisfactory diagnostic performance, we anticipate that the proposed EGSVM will be a reliable and reproducible tool for the classification of thermal images.

The EGSVM-based infrared thermal imaging system has several advantages as a screening and diagnostic tool for the detection of cervical lymph node metastasis from oral cancer. First, infrared thermal imaging is completely risk-free. It passively captures thermal radiation emitted by the human body, as there is no need for ionized radiation, contrast agents or any invasive procedures. Second, compared with other medical imaging examinations, IR examination is inexpensive due to the low-cost IR camera that is used. In addition, IR image capture and automatic image analysis can be completed in less than one minute each, allowing results to be obtained quickly.

This is a prospective pilot study, and there are still limitations. First, this is a patient-based diagnosis of cervical lymph node metastasis from oral cavity cancer. Metastatic lymph nodes and the vascular abnormalities they cause can both result in deviations in heat, which provides the basis for the use of infrared imaging. While we can locate the abnormal area in two-dimensional space, it is difficult to precisely identify the lymph nodes within this area. Due to its limitations in identifying the exact anatomical localization of lesions, this method is currently more suitable for use as a screening tool. We anticipate that the EGSVM-based infrared thermal imaging system will play a role as a fast screening tool for detecting of cervical lymph node metastasis from oral cavity cancer in resource-limited environments. Second, in this study, we extracted only one feature for automatic classification; the performance of the model can be improved with additional useful features. Additionally, more patients are needed to evaluate the diagnostic performance of this method in a multi-center clinical trial before it can be widely used for the detection of cervical lymph node metastasis from oral cavity cancer.
