# Author Correction: Detection of cervical lymph node metastasis from oral cavity cancer using a non-radiating, noninvasive digital infrared thermal imaging system

**DOI:** 10.1038/s41598-018-28632-2

**Published:** 2018-07-10

**Authors:** Fan Dong, Chuansibo Tao, Ji Wu, Ying Su, Yuguang Wang, Yong Wang, Chuanbin Guo, Peijun Lyu

**Affiliations:** 1 National Engineering Laboratory for Digital and Material Technology of Stomatology, Peking University School and Hospital of Stomatology, 22 Zhongguancun Avenue South, Haidian District, Beijing, 100081 P. R. China; 2 Center of Digital Dentistry, Peking University School and Hospital of Stomatology, 22 Zhongguancun Avenue South, Haidian District, Beijing, 100081 P. R. China; 3 Department of Prosthodontics, Peking University School and Hospital of Stomatology, 22 Zhongguancun Avenue South, Haidian District, Beijing, 100081 P. R. China; 4 Research Center of Engineering and Technology for Digital Dentistry of Ministry of Health, 22 Zhongguancun Avenue South, Haidian District, Beijing, 100081 P. R. China; 5 Beijing Key Laboratory of Digital Stomatology, 22 Zhongguancun Avenue South, Haidian District, Beijing, 100081 P. R. China; 6 Department of Oral and Maxillofacial Surgery, Peking University School and Hospital of Stomatology, 22 Zhongguancun Avenue South, Haidian District, Beijing, 100081 P. R. China; 7 Tsinghua-Rohm Electronic Engineering Hall 8-301, Tsinghua University, Beijing, 100084 P. R. China

Correction to: *Scientific Reports* 10.1038/s41598-018-24195-4, published online 08 May 2018

The original version of this Article contained an error in Affiliation 1, which was incorrectly given as ‘National Engineering Laboratory for Digital and Material Technology of Stomatology, 22 Zhongguancun Avenue South, Haidian District, Beijing, 100081, P. R. China’. The correct affiliation is listed below:

‘National Engineering Laboratory for Digital and Material Technology of Stomatology, Peking University School and Hospital of Stomatology, 22 Zhongguancun Avenue South, Haidian District, Beijing, 100081, P. R. China’

This error has now been corrected in the HTML and PDF versions of the Article.

